# Supplementary material for: Loss of consciousness reduces the stability of brain hubs and the heterogeneity of brain dynamics
Source: Commun Biol. 2021 Sep 6;4:1037. doi: 10.1038/s42003-021-02537-9 (PMC8421429; doi:10.1038/s42003-021-02537-9)
Supplement: Supplementary file 2 — Supplementary Information [file 42003_2021_2537_MOESM2_ESM.pdf]

Loss of consciousness reduces the stability of brain hubs and the heterogeneity of brain dynamics

Ane López-González[1,*,†], Rajanikant Panda[2,3,†], Adrián Ponce-Alvarez[1], Gorka Zamora-López[1], Anira Escrichs[1], Charlotte Martial[2,3], Aurore Thibaut[2,3], Olivia Gosseries[2,3], Morten L. Kringelbach[4,5,6], Jitka Annen[2,3], Steven Laureys[2,3,‡], and Gustavo Deco[1,7,‡]

[1]Computational Neuroscience Group, Center for Brain and Cognition, Universitat Pompeu Fabra, Barcelona, Spain

[2]GIGA-Consciousness, Coma Science Group, University of Liège, Liège, Belgium

[3]Centre du Cerveau[2], University Hospital of Liège, Liège, Belgium

[4]Department of Psychiatry, University of Oxford, Oxford OX3 7JX, United Kingdom

[5]Center for Music in the Brain, Department of Clinical Medicine, Aarhus University, DK-8000 Aarhus C, Denmark

[6]Life and Health Sciences Research Institute, School of Medicine, University of Minho, 4710-057, Braga, Portugal

[7]Institució Catalana de la Recerca i Estudis Avançats (ICREA), Passeig Lluís Companys 23, 08010 Barcelona, Spain

[†]These authors contributed equally to this work.

[‡]These authors jointly supervised this work.

[*]Correspondence should be addressed to A.LG (email: ane.lopez@upf.edu)

Supplementary Figures and Tables

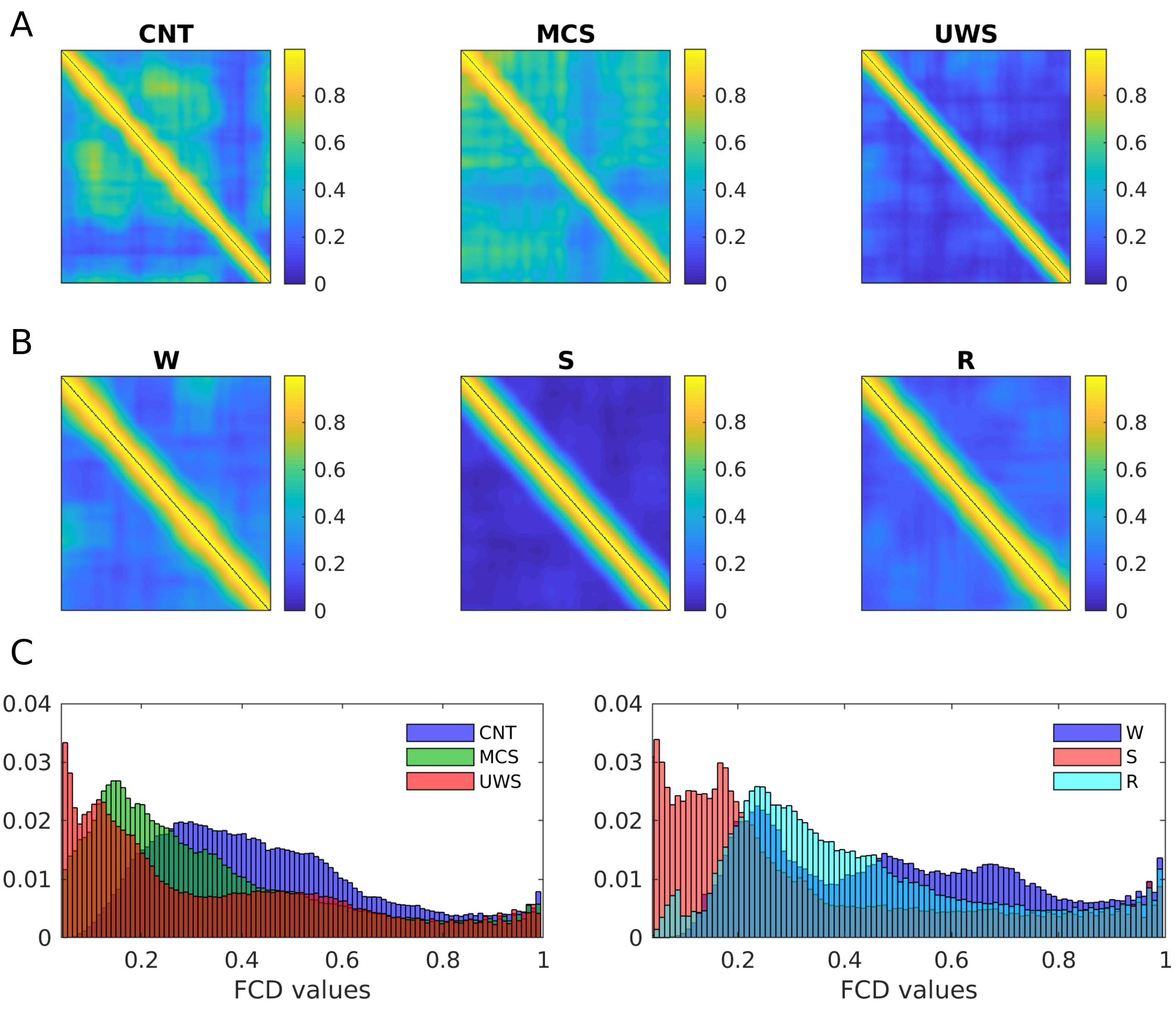

Supplementary Figure S1: **Functional Dynamical Connectivity (FCD) matrices and distributions of their values.** The FCD matrices were calculated based on the cosine similarity between phase difference matrices at different time windows. **A)** Examples of the FCD for individual healthy subjects and DOC patients. **B)** FCD matrices for individual subjects during wakefulness, sedation and recovery from propofol sedation. **C)** The distribution of the upper diagonal elements of the FCD matrix, for all the subjects in each group. The distribution of the conscious states (controls and W) was sparser and shifted towards higher values compared to low-level states of consciousness.

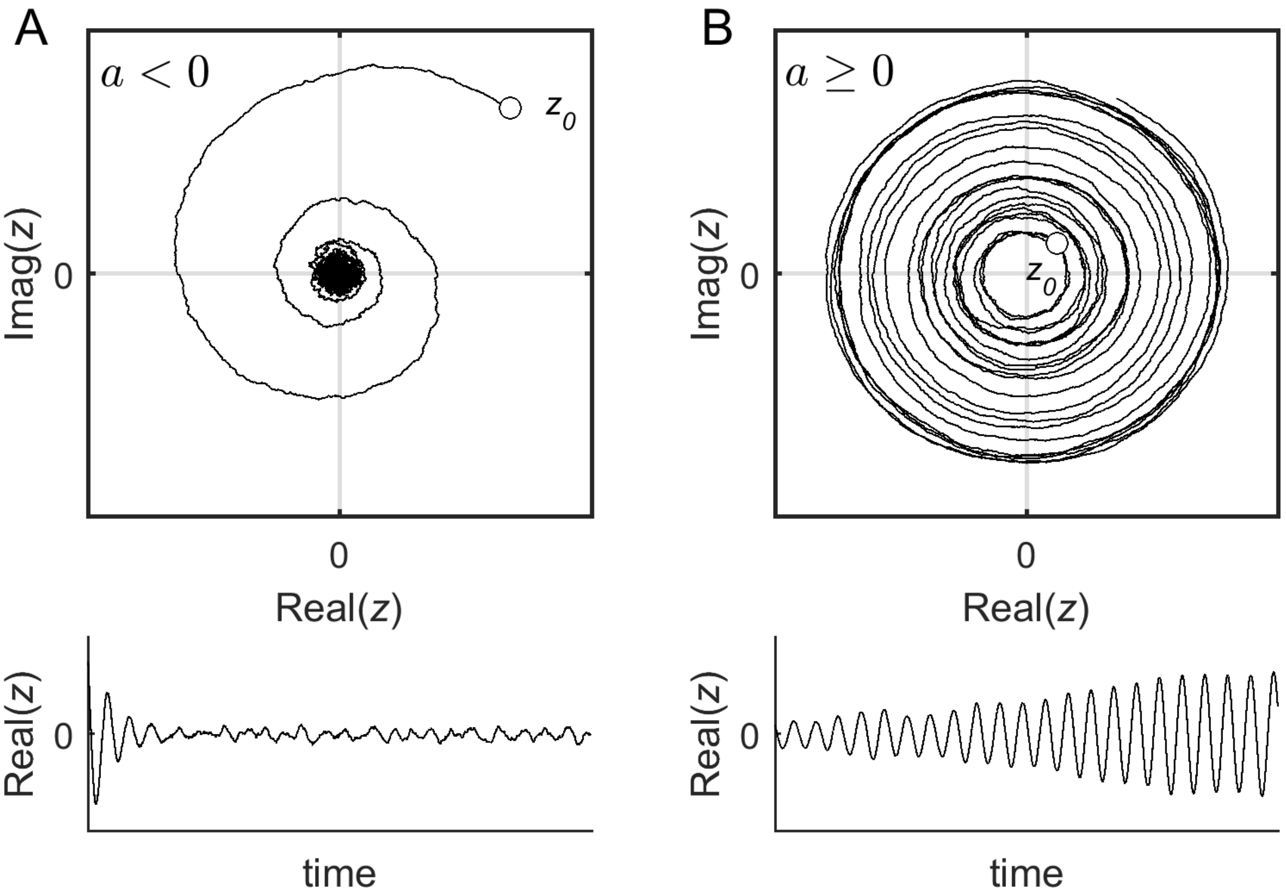

Supplementary Figure S2: **Phase space for an example of a single Hopf oscillator.** **A)** Subcritical Hopf oscillator ($a < 0$). Top: In this regime, a stable spiral, or focus, exists at $\boldsymbol{z} = 0$. The system relaxes towards the focus with damped oscillations. In the presence of noise, however, the system fluctuates around the focus, thus producing noise-induced oscillations. $\boldsymbol{z_0} = \boldsymbol{z}(t = 0)$ indicates the initial condition. Bottom: temporal evolution of $Real(z)$. **B)** Supercritical Hopf bifurcation ($a \geq 0$). Top: In this regime, the focus at $\boldsymbol{z} = 0$ becomes unstable and a stable limit-cycle appears, thus producing autonomous or self-sustained oscillations. Bottom: temporal evolution of $Real(z)$.

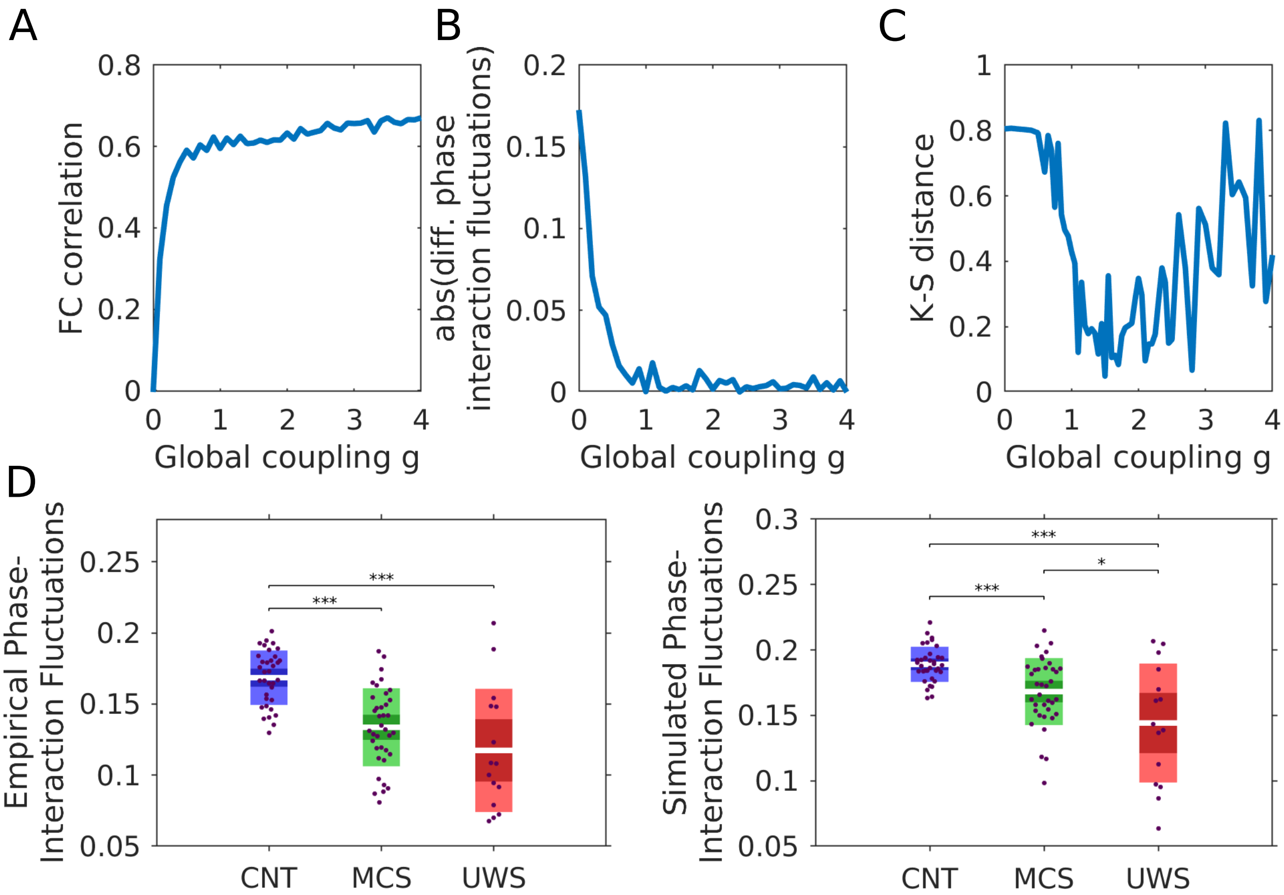

Supplementary Figure S3: **Goodness of fit of the whole-brain computational model computed by using different measures.** **A)** Pearson correlation between the empirical and simulated functional connectivity (FC) matrices as a function of parameter g. **B)** Absolute difference between the empirical and simulated phase-interaction fluctuations as a function of g. **C)** Kolmogorov-Smirnov distance between the empirical and simulated FCD distributions. **D)** The optimal value of g was obtained using the KS-distance between the empirical and simulated FCD distributions. We verified that, for the obtained values of g in each condition, the differences in the phase interaction fluctuations observed in the data were preserved in the model. For this we used the SC matrix of individual subjects and the parameter g was fixed for each group. Boxplots represent the mean of the measures' values with a 95% confidence interval (dark) and 1 SD (light). Differences between groups were assessed using one-way ANOVA followed by FDR p-value correction. *: $p < 0.05$; **: $p < 0.01$; ***: $p < 0.001$.

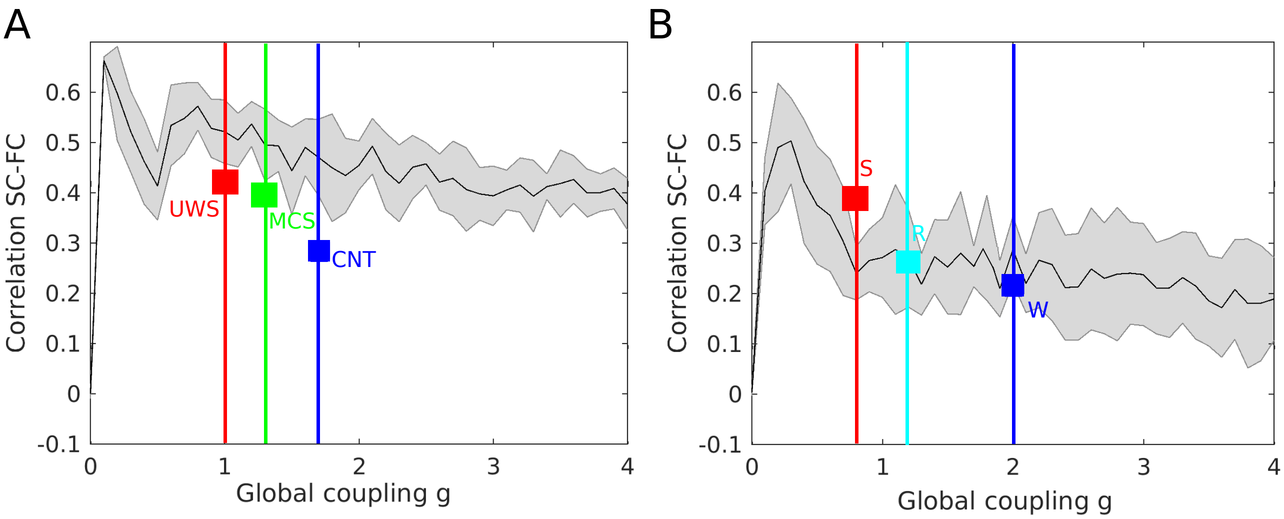

Supplementary Figure S4: **Empirical and model correlations between the SC and FC matrices for each group.** In **A)** and **B)** the black line corresponds to the correlation between the empirical SC and the FC simulated with the whole-brain model learned from the different experimental groups, as a function of g. The shaded area corresponds to the standard error of the values obtained for different simulations. The curve shows a peak for small g and then it decreases slowly as g increases. In **A)** the lines are located in the corresponding optimal global coupling g for each brain state. In **B)** the lines correspond to the states for the propofol anaesthesia dataset. For all cases, the values correspond to the optimal g extracted from the homogeneous model (depicted in Fig. 2). In **A)** and **B)** the squares correspond to the empirical values of the empirical SC and FC correlation. Both empirically and using the model, we observed a shift to smaller values in the SC-FC correlation while the level of consciousness decreases.

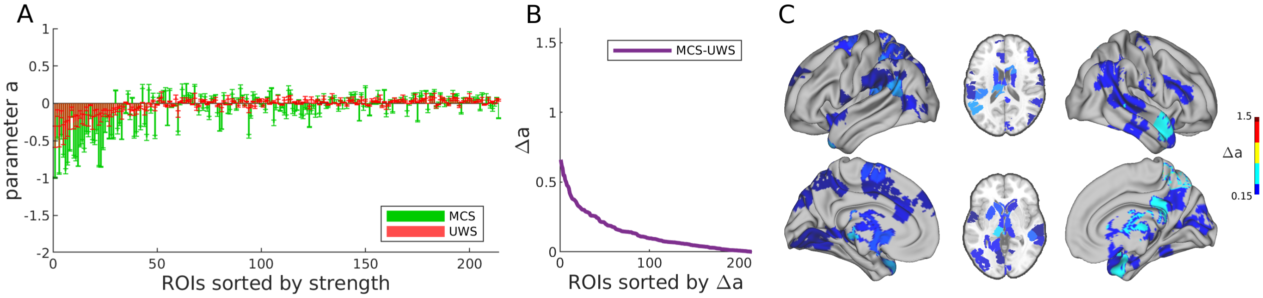

Supplementary Figure S5: **Comparison between the local bifurcation parameters of the whole-brain model between DOC patients (MCS and UWS). A)**Bars indicate the mean ± standard deviation across simulations of estimated bifurcation model parameters for each of the 214 nodes (sorted by node strength). Green bars correspond to MCS and red bars to UWS. **B** Ranked absolute parameter difference, Δa, for each the comparison between the two groups. **C)** Spatial distribution of the most altered bifurcation parameter values.

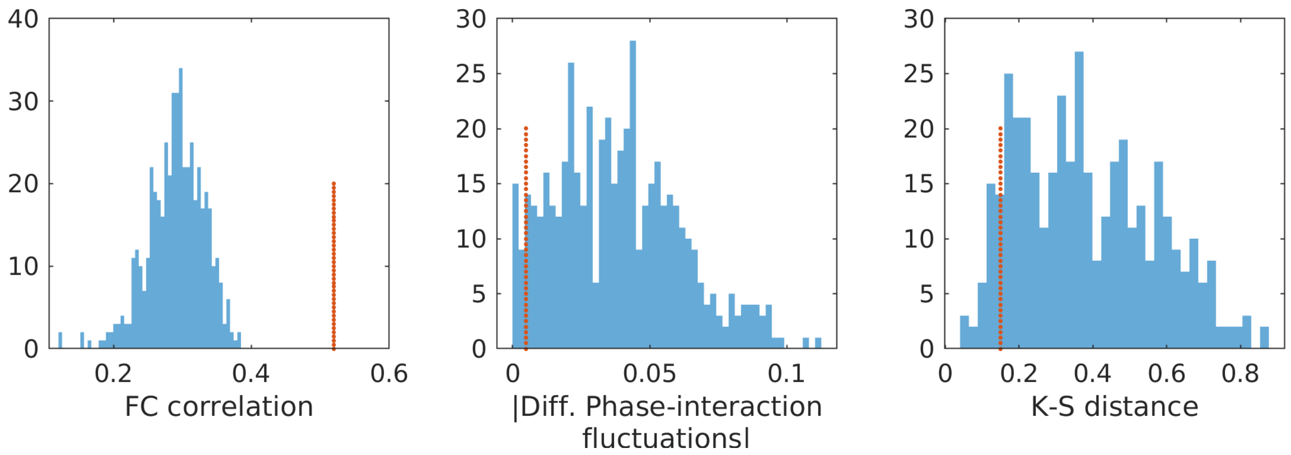

Supplementary Figure S6: **Goodness of fit of the heterogeneous model compared to models shuffling the order of the a's.** Goodness of fit for the Pearson correlation of the FC matrices, the absolute difference in phase-interaction fluctuations and the Kolmogorv-Smirnov distance between the FCD matrices. The blue histograms correspond to the fitting after shuffling the labels of the a_i values and the red line corresponds to the mean fitting of the heterogeneous model.

38% Superior frontal gyrus, orbital part R / 25% Middle frontal gyrus, orbital part R
42% Gyrus rectus R / 19% Olfactory cortex R
69% Superior frontal gyrus, orbital part R
44% Middle frontal gyrus R/ 31% Anterior cingulate and paracingulate gyri
34% Insula R/ 22% Lenticular nucleus, putamen R
56% Temporal pole: middle temporal gyrus R/ 20% Fusiform gyrus R
67% Inferior temporal gyrus R
55% Fusiform gyrus R/ 41% Inferior temporal gyrus R
33% Fusiform gyrus R/ 27% Inferior temporal gyrus R
70% Inferior temporal gyrus R
42% Median cingulate and paracingulate gyri R
50% Hippocampus R/ 9% ParaHippocampal gyrus R
30% Hippocampus R/ 15% Amygdala R
41% Thalamus R/ 8% Hippocampus R
52% Thalamus R
40% Superior frontal gyrus, orbital part L/ 34%Middle frontal gyrus, orbital part L
58% Paracentral Lobule L / 35% Precuneus L
61% Inferior temporal gyrus L
55% Temporal pole: superior temporal gyrus L/ 42% Temporal pole: middle temporal gyrus L
45% emporal pole: middle temporal gyrus L/ 25% Fusiform gyrus L
87% Inferior temporal gyrus L
65% Inferior temporal gyrus L
59% Calcarine fissure and surrounding cortex L/ 7% Lingual gyrus L
55% Posterior cingulate gyrus L / 21% Precuneus L
31% Amygdala L / 18%Temporal pole: superior temporal gyrus L
42% Hippocampus L/ 5% Thalamus L
30% Hippocampus L / 21% ParaHippocampal gyrus L
84% Lenticular nucleus, putamen L
53% Thalamus L
51% Thalamus L / 2% Caudate nucleus L

Supplementary Table S1: **ROIs with the highest absolute difference in the bifurcation parameters when comparing normal wakefulness in healthy controls and MCS.** All the ROIs showed an absolute difference greater than the threshold given by the sum of the mean and standard deviation of the absolute differences corresponding to all the ROIs. The percentage corresponds to the covered part of the ROI in the Automated Anatomical Labeling (AAL) parcellation code.

42 % Gyrus rectus R / 19 % Olfactory cortex R
69 % Superior frontal gyrus, orbital part R
34 % Insula R/ 22 % Lenticular nucleus, putamen R
56 % Temporal pole: middle temporal gyrus R/ 20 % Fusiform R
67 % Inferior temporal gyrus R
48 % Fusiform R/ 38 % Inferior temporal gyrus R
55 % Fusiform R/ 41 % Inferior temporal gyrus R
33 % Fusiform R/ 27 % Inferior temporal gyrus R
44 % Calcarine fissure and surrounding cortex R/ 6 % Lingual gyrus R
28 % Amygdala R/ 27 % Temporal pole: superior temporal gyrus R
50 % Hippocampus R/ 9 % Parahippocampal gyrus R
30 % Hippocampus R / 15 % Amygdala R
62 % Caudate nucleus R
46 % Caudate nucleus R / 6 % Thalamus R
84 % Lenticular nucleus, putamen R
36 % Thalamus R/ 9 % Lingual gyrus R
41 % Thalamus R / 8 % Hippocampus R
51 % Inferior frontal gyrus, orbital part L / 31 % Superior frontal gyrus, orbital part L
56 % Gyrus rectus L/ 13 % Olfactory cortex L
34 % Insula L/ 18 % Superior temporal gyrus L
61 % Inferior temporal gyrus L
45 % Temporal pole: middle temporal gyrus L/ 25 % Fusiform L
46 % Inferior temporal gyrus L/ 41 % Fusiform L
87 % Inferior temporal gyrus L
65 % Inferior temporal gyrus L
59 % Fusiform L/ 21 % Inferior temporal gyrus L
59 % Calcarine L/ 7 % Lingual gyrus L
55 % Posterior cingulate gyrus L/ 21 % Precuneus L
42 % Hippocampus L/ 5 % Thalamus L
30 % Hippocampus L/ 21 % Parahippocampal gyrus L
28 % Caudate nucleus L/ 15 % Olfactory cortex L
52 % Caudate nucleus L/ 2 % Thalamus L
51 % Thalamus L/ 2 % Caudate nucleus L

Supplementary Table S2: **ROIs with the highest absolute difference in the bifurcation parameters when comparing normal wakefulness in healthy controls and UWS.** All the ROIs showed an absolute difference greater than the threshold given by the sum of the mean and standard deviation of the absolute differences corresponding to all the ROIs. The percentage corresponds to the covered part of the ROI in the Automated Anatomical Labeling (AAL) parcellation code.

55 % Fusiform R / 41 % Inferior temporal gyrus R
44 % Calcarine fissure and surrounding cortex R/ 6 % Lingual gyrus R
50 % Hippocampus R / 9 % Parahippocampal gyrus R
30 % Hippocampus R / 15 % Amygdala R
62 % Caudate nucleus R
46 % Caudate nucleus R/ 6 % Thalamus R
57 % Caudate nucleus R/ 11 % Olfactory cortex R
84 % Lenticular nucleus, putamen R
41 % Thalamus R/ 8 % Hippocampus R
83 % Inferior temporal gyrus L
59 % Calcarine fissure and surrounding cortex L/ 7 % Lingual gyrus L
37 % Median cingulate and paracingulate gyri L/ 36 % Anterior cingulate and paracingulate gyri L
55 % Posterior cingulate gyrus L / 21 % Precuneus L
42 % Hippocampus L / 5 % Thalamus L
30 % Hippocampus L/ 21 % Parahippocampal gyrus L
46 % Hippocampus L / 19 % Inferior temporal gyrus L
28 % Caudate nucleus L / 15 % Olfactory cortex L
52 % Caudate nucleus L / 2 % Thalamus L
51 % Thalamus L / 2 % Caudate nucleus L

Supplementary Table S3: **ROIs with the highest absolute difference in the bifurcation parameters when comparing normal wakefulness, W and sedation, S.** All the ROIs showed an absolute difference greater than the threshold given by the sum of the mean and standard deviation of the absolute differences corresponding to all the ROIs. The percentage corresponds to the covered part of the ROI in the Automated Anatomical Labeling (AAL) parcellation code.

74 % Postcentral gyrus R
55 % Fusiform R/ 41 % Inferior temporal gyrus R
33 % Fusiform R / 27 % Inferior temporal gyrus R
70 % Inferior temporal gyrus R
67 % Middle occipital gyrus R
50 % Hippocampus R / 9 % Parahippocampal gyrus R
46 % Caudate nucleus R / 6 % Thalamus R
84 % Lenticular nucleus, putamen R
41 % Thalamus R / 8 % Hippocampus R
40 % Superior frontal gyrus,medial L/ 30 % Superior frontal gyrus, dorsolateral L
61 % Postcentral gyrus L
42 % Rolandic operculum L/ 39 % Insula L
83 % Inferior temporal gyrus L
63 % Inferior temporal gyrus L
37 % Median cingulate and paracingulate gyri L /<br>36 % Anterior cingulate and paracingulate gyri L
39 % Median cingulate and paracingulate gyri L/ 22 % Posterior cingulate gyrus L
55 % Posterior cingulate gyrus L / 21 % Precuneus L
42 % Hippocampus L/ 5 % Thalamus L
30 % Hippocampus L/ 21 % Parahippocampal gyrus L
55 % Hippocampus L / 13 % Parahippocampal gyrus L
28 % Caudate nucleus L/ 15 % Olfactory cortex L

Supplementary Table S4: **ROIs with the highest absolute difference in the bifurcation parameters when comparing normal wakefulness, W and recovery from propofol-sedation R.** All the ROIs showed an absolute difference greater than the threshold given by the sum of the mean and standard deviation of the absolute differences corresponding to all the ROIs. The percentage corresponds to the covered part of the ROI in the Automated Anatomical Labeling (AAL) parcellation code.

41 % Thalamus R / 8 % Hippocampus R	44 % Frontal Medial L / 43 % Superior frontal gyrus, dorsolateral L
55 % Middle temporal gyrus L/ 27 % Angular gyrus L	50 % Superior frontal gyrus,medial L/ 47 % Anterior cingulate and paracingulate gyri L
80 % Median cingulate and paracingulate gyri R	42 % Rolandic operculum L / 39 % Insula L
51 % Thalamus L / 2 % Caudate nucleus L	75 % Precuneus L
42 % Hippocampus L / 5 % Thalamus L	71 % Fusiform L
66 % Median cingulate and paracingulate gyri L	57 % Inferior parietal gyrus L / 36 % Supramarginal gyrus L
74 % Supplementary motor area R	82 % Middle temporal gyrus L
58 % Fusiform L / 36 % Inferior temporal gyrus L	54 % Postcentral gyrus L/ 28 % Inferior parietal gyrus L
44 % Superior temporal gyrus L / 26 % Rolandic operculum L	50 % Hippocampus R/ 9 % Parahippocampal gyrus R
81 % Anterior cingulate and paracingulate gyri R	88 % Lingual gyrus L
52 % Median cingulate and paracingulate gyri L/ 45 % Supplementary motor area L	60 % Supplementary motor area R/ 22 % Median cingulate and paracingulate gyri R
44 % Frontal Med VMPFC R/ 31 % Anterior cingulate and paracingulate gyri R	46 % Inferior frontal gyrus, orbital part L / 36 % Insula L
53 % Thalamus L/ 0 % Thalamus R	55 % Posterior cingulate gyrus L/ 21 % Precuneus L
33 % Anterior cingulate and paracingulate gyri L / 23 % Gyrus rectus L	52 % Superior frontal gyrus, dorsolateral R/ 41 % Superior frontal gyrus,medial R
57 % Gyrus rectus R / 23 % Frontal Med R	67 % Calcarine fissure and surrounding cortex L
65 % Inferior temporal gyrus L	64 % Middle temporal gyrus R
52 % Thalamus R	62 % Temporal pole: middle temporal gyrus R
46 % Caudate nucleus R/ 6 % Thalamus R	57 % Precuneus R/ 32 % Calcarine fissure and surrounding cortex R
56 % Median cingulate and paracingulate gyri R/ 26 % Anterior cingulate and paracingulate gyri R	63 % Inferior temporal gyrus L
80 % Precuneus R	30 % Hippocampus L/ 21 % Parahippocampal gyrus L
54 % Frontal Med VMPFC L/ 31 % Anterior cingulate and paracingulate gyri L	42 % Gyrus rectus R/ 19 % Olfactory cortex R
56 % Gyrus rectus L/ 13 % Olfactory cortex L	62 % Middle temporal gyrus L
49 % Inferior parietal gyrus L/ 38 % Postcentral gyrus L	49 % Inferior occipital gyrus L/ 33 % Fusiform gyrus L
81 % Median cingulate and paracingulate gyri R	36 % Thalamus R / 9 % Lingual R
47 % Supramarginal gyrus R/ 35 % Postcentral gyrus R	87 % Angular gyrus R
52 % Caudate nucleus L/ 2 % Thalamus L	51 % Lingual gyrus R/ 33 % Fusiform gyrus R
65 % Cuneus R	50 % Precental gyrus L/ 30 % Inferior frontal gyrus, opercular part L
62 % Caudate nucleus R	57 % Caudate nucleus R/ 11 % Olfactory cortex R
40 % Fusiform gyrus L/ 31 % Lingual gyrus L	45 % Precuneus L/ 34 % Calcarine fissure and surrounding cortex L
50 % Anterior cingulate and paracingulate gyri L/ 39 % Superior frontal gyrus, medial L	46 % Supramarginal gyrus R/ 34 % Inferior parietal gyrus R
59 % Inferior frontal gyrus, orbital part R/ 20 % Insula R	47 % Postcentral gyrus R/ 23 % Inferior parietal gyrus R
72 % Hippocampus R	34 % Calcarine fissure and surrounding cortex L/ 26 % Middle occipital gyrus L
46 % Thalamus L/ 1 % Lingual gyrus L	61 % Inferior temporal gyrus L

Supplementary Table S5: **ROIs that show a significant decrease ($p < 0.05$) when comparing the values of the strength obtained from the control subjects and the MCS patients.** Statistics were assessed using Ranksum Wilconsom test, followed by FDR p-value correction. The percentage corresponds to the covered part of the ROI in the Automated Anatomical Labeling (AAL) parcellation code.

41 % Thalamus R/ 8 % Hippocampus R	47 % Supramarginal gyrus R/ 35 % Postcentral R
51 % Thalamus L/ 2 % Caudate nucleus L	61 % Inferior temporal gyrus L
53 % Thalamus L/ 0 % Thalamus R	49 % Inferior occipital gyrus L/ 33 % Fusiform gyrus L
50 % Precental gyrus L/ 30 % Inferior frontal gyrus, opercular part L	48 % Fusiform gyrus R/ 38 % Inferior temporal gyrus R
80 % Precuneus R	70 % Superior parietal gyrus R
52 % Thalamus R	58 % Supplementary motor area R/<br>42 % Superior frontal gyrus, dorsolateral R
42 % Hippocampus L/ 5 % Thalamus L	65 % Cuneus R
75 % Precuneus L	36 % Thalamus R/ 9 % Lingual gyrus R
33 % Anterior cingulate and paracingulate gyri L/ 23 % Gyrus rectus L	66 % Median cingulate and paracingulate gyri L
63 % Postcentral gyrus L 46 % Inferior frontal gyrus, orbital part L	36 % Insula L
54 % Postcentral gyrus L/ 28 % Inferior parietal gyrus L	63 % Inferior frontal gyrus, triangular gyrus part L
80 % Median cingulate and paracingulate gyri R	46 % Inferior temporal gyrus L / 41 % Fusiform gyrus L
46 % Caudate nucleus R/ 6 % Thalamus R	59 % Fusiform gyrus L/ 21 % Inferior temporal gyrus L
52 % Caudate nucleus L/ 2 % Thalamus L	45 % Inferior frontal gyrus, triangular part R/<br>28 % Inferior frontal gyrus, orbital part R
44 % Superior frontal gyrus,medial L/<br>43 % Superior frontal gyrus, dorsolateral L	66 % Inferior temporal gyrus R
56 % Gyrus rectus L/ 13 % Olfactory cortex L	66 % Middle frontal gyrus L
49 % Inferior parietal gyrus L/ 38 % Postcentral gyrus L	52 % Superior frontal gyrus, dorsolateral R/<br>41 % Superior frontal gyrus,medial R
62 % Caudate nucleus R	57 % Gyrus rectus R / 23 % Frontal Med R
62 % Middle temporal gyrus L	56 % Median cingulate and paracingulate gyri R<br>/ 26 % Anterior cingulate and paracingulate gyri R
81 % Median cingulate and paracingulate gyri R	53 % Fusiform gyrus R/ 16 % Lingual gyrus R
44 % Medial frontal gyrus R/<br>31 % Anterior cingulate and paracingulate gyri R	50 % Hippocampus R/ 9 % Parahippocampal gyrus R
55 % Middle temporal gyrus L/ 27 % Angular gyrus L	52 % Median cingulate and paracingulate gyri L/<br>45 % Supplementary motor area L
72 % Hippocampus R	66 % Postcentral gyrus R
57 % Precuneus R/ 32 % Calcarine fissure and surrounding cortex R	47 % Postcentral gyrus R/ 23 % Inferior parietal gyrus R
58 % Fusiform gyrus L/ 36 % Inferior temporal gyrus L	28 % Caudate nucleus L/ 15 % Olfactory cortex L
40 % Fusiform gyrus L/ 31 % Lingual L	63 % Postcentral gyrus R
44 % Inferior frontal gyrus, opercular part R/<br>34 % Inferior frontal gyrus, triangular part R	55 % Posterior cingulate gyrus L/ 21 % Precuneus L
57 % Inferior parietal gyrus L/ 36 % Supramarginal gyrus L	28 % Amygdala R / 27 % Temporal pole: superior temporal gyrus R
59 % Inferior frontal gyrus, orbital part R/ 20 % Insula R	62 % Temporal pole: middle temporal gyrus R
46 % Thalamus L/ 1 % Lingual gyrus L	81 % Supplementary motor area L
42 % Superior occipital gyrus R/ 26 % Cuneus R	34 % Insula R/ 22 % Lenticular nucleus, putamen R
51 % Lingual gyrus R/ 33 % Fusiform gyrus R	42 % Rolandic operculum L / 39 % Insula L
57 % Superior frontal gyrus,medial L/<br>41 % Superior frontal gyrus, dorsolateral L	76 % Calcarine fissure and surrounding cortex R
75 % Middle frontal gyrus L	51 % Inferior frontal gyrus, orbital part L / 31 % Frontal SomeOrb L
48 % Fusiform gyrus R/ 29 % Inferior temporal gyrus R	87 % Caudate nucleus L
44 % Superior temporal gyrus L/ 26 % Rolandic operculum L	50 % Superior frontal gyrus,medial L/<br>47 % Anterior cingulate and paracingulate gyri L
71 % Fusiform gyrus L	55 % Hippocampus L / 13 % ParaHippocampal L
67 % Calcarine fissure and surrounding cortex L	65 % Inferior temporal gyrus L
88 % Lingual gyrus L	84 % Middle frontal gyrus R
19 % Lenticular nucleus, putamen R/ 14 % Caudate nucleus R	52 % Precental gyrus R/ 22 % Inferior frontal gyrus, opercular part R
54 % Frontal Med VMPFC L/<br>31 % Anterior cingulate and paracingulate gyri L	78 % Lingual gyrus R
81 % Anterior cingulate and paracingulate gyri R	73 % Middle occipital gyrus L
30 % Hippocampus L/ 21 % Parahippocampal gyrus L	74 % Postcentral gyrus R
39 % Fusiform gyrus R/ 25 % Inferior occipital gyrus R	44 % Calcarine fissure and surrounding cortex R/ 6 % Lingual gyrus R
56 % Temporal pole: middle temporal gyrus R/ 20 % Fusiform gyrus R	33 % Fusiform gyrus R / 27 % Inferior temporal gyrus R
59 % Angular gyrus L/ 26 % Inferior parietal gyrus L	82 % Middle temporal gyrus L
67 % Inferior temporal gyrus R	

Supplementary Table S6: **ROIs that show a significant decrease ($p < 0.05$) when comparing the values of the strength obtained from the control subjects and UWS patients.** Statistics were assesed using Ranksum Wilconsom test, followed by FDR p-value correction. The percentage corresponds to the covered part of the ROI in the Automated Anatomical Labeling (AAL) parcellation code.

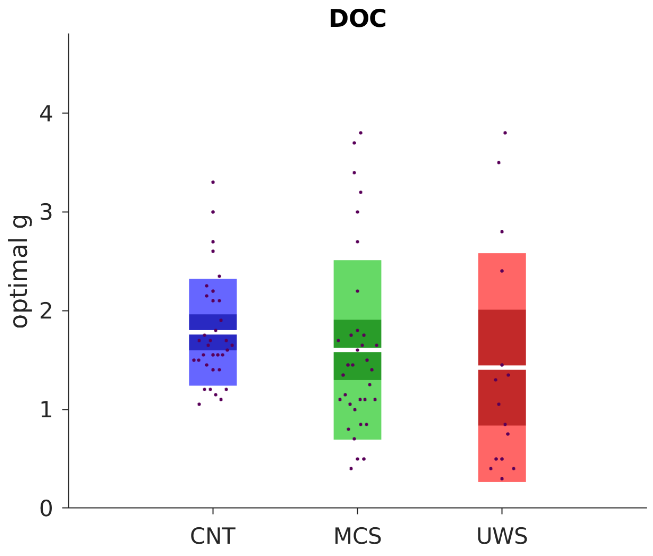

Supplementary Figure S7: **Whole-brain model global coupling parameter fitting for the individual SC.** Optimal global coupling g for each of the subjects of the DOC dataset using the individual SC to set the interactions between model nodes. One-way-ANOVA p-value: $p_{CNT-MCS} = 0.665$, $p_{CNT-UWS} = 0.446$ and $p_{UWS-MCS} = 0.878$. Boxplots represent the mean of the measures' values with a 95% confidence interval (dark) and 1 SD (light).

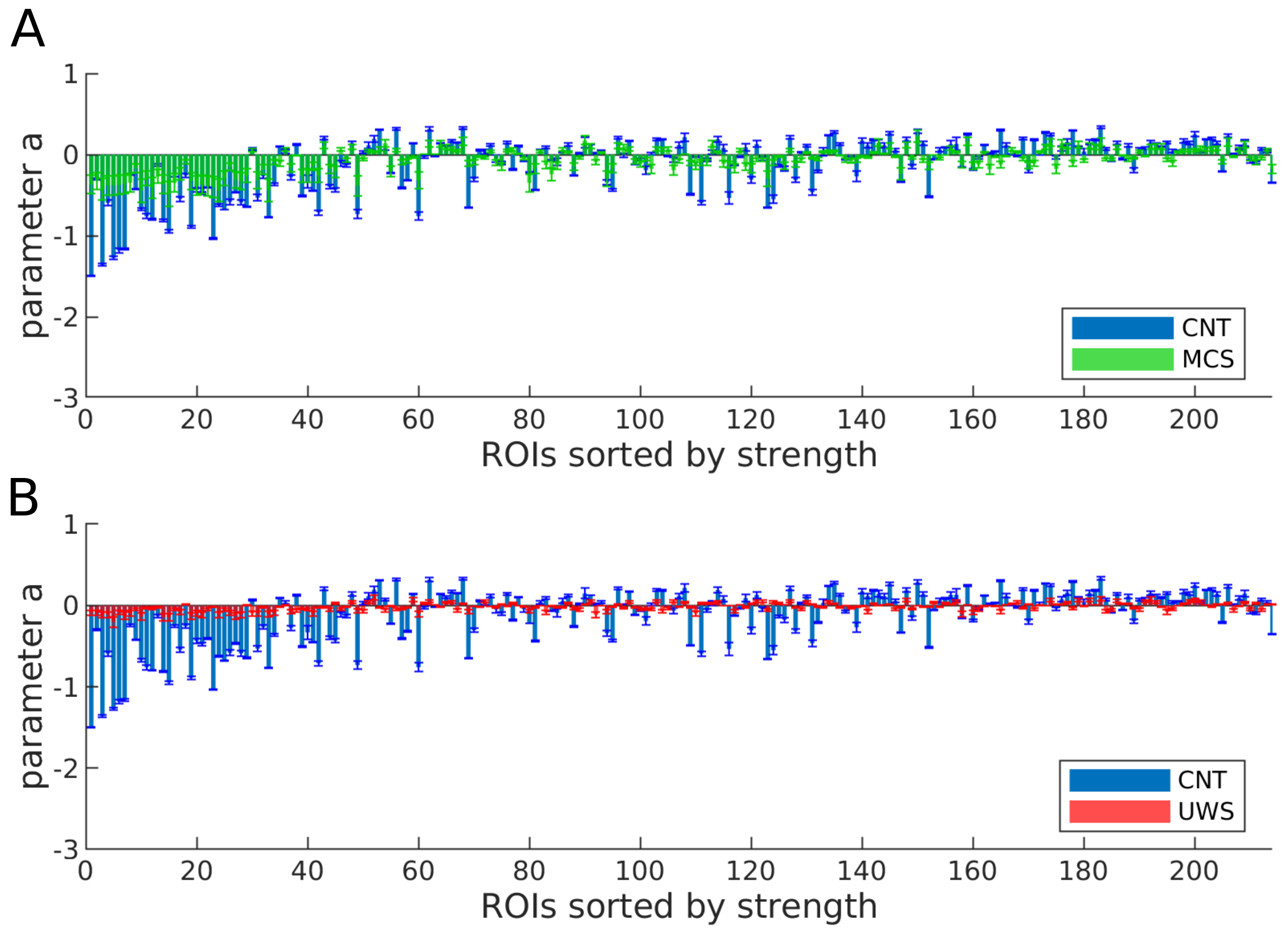

Supplementary Figure S8: **Local bifurcation parameters of the whole-brain model when using the as SC the patients average SC. A-B)** Bars indicate the mean ± standard error of estimated bifurcation model parameters for each of the 214 nodes (sorted by node strength of the controls SC). Each DOC group results were compared to the wakefulness in heakthy controls.

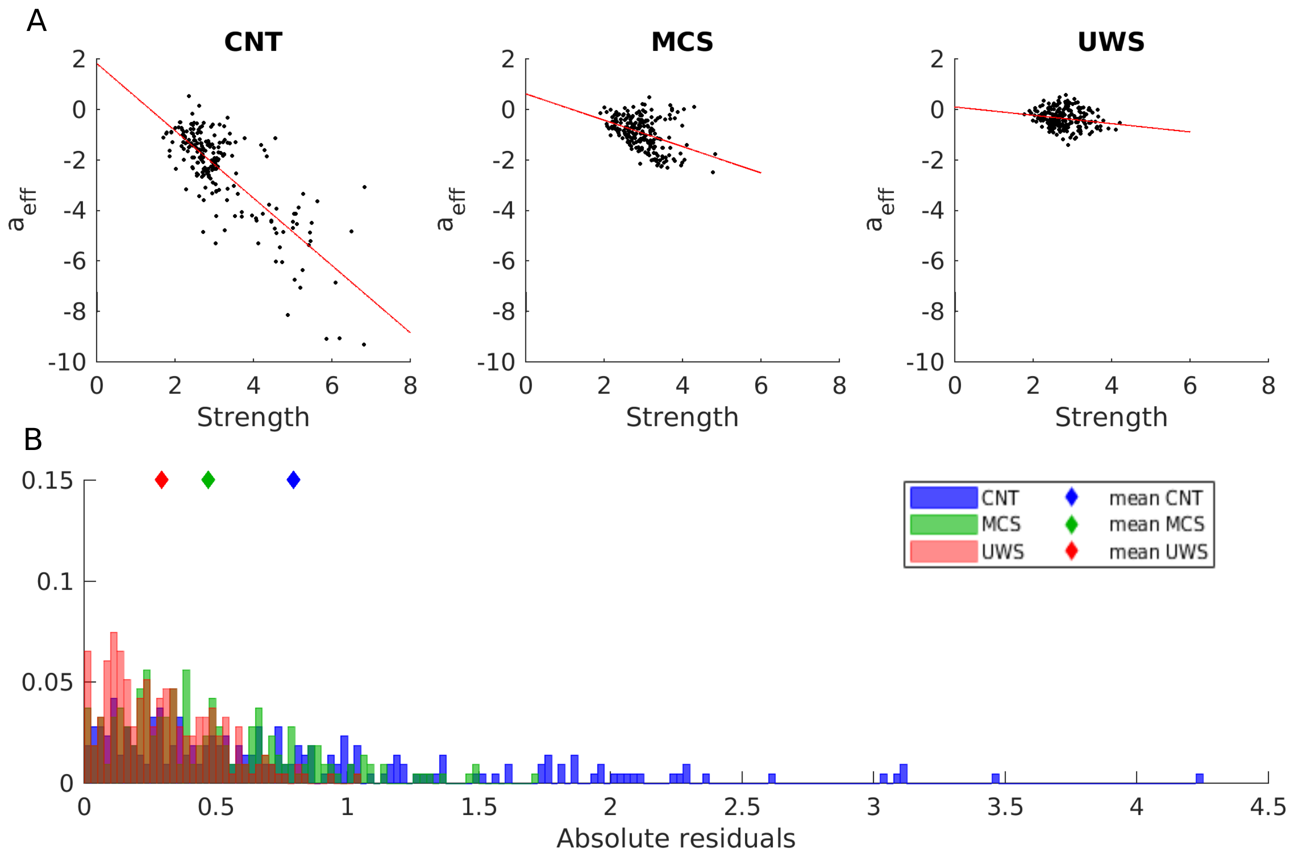

Supplementary Figure S9: **Relation between the a_j^{eff} and connectivity strength of the SC characterizing each group.** **A)** The effective local bifurcation parameters, a_j^{eff}, were estimated using the heterogeneous model and group SC. The g was fixed in all cases using the mean showed in Fig. 2 E. The obtained parameters were compared to the strengths of the nodes S_j in each group SC. The red lines indicate the linear fits. **B)** Distribution of the absolute residuals of each node given by the squared difference between the value of a_j^{eff} and the estimated linear relationship between a_j^{eff} and S_j, for each group.

Condition	Etiology	TSI	Age	Gender	Auditory	Visual	Motor	Verbal	Communication	Arousal	Total CRS-R
MCS 1	TBI	3034	34	F	3	3	2	2	0	2	12
MCS 2	TBI	1294	40	F	2	3	2	2	0	2	11
MCS 3	CVA	13	62	M	0	3	2	1	0	1	7
MCS 4	TBI	589	30	M	3	2	2	2	0	1	10
MCS 5	TBI	28	65	M	3	4	3	1	0	2	13
MCS 6	Haemorrhage	17	83	M	3	0	2	1	0	0	6
MCS 7	TBI	521	28	M	1	3	2	2	0	2	10
MCS 8	Epilepsy	20	52	M	3	3	2	2	1	2	13
MCS 9	Haemorrhage	43	67	M	2	3	5	2	0	2	14
MCS 10	TBI	533	47	M	3	5	2	1	0	2	13
MCS 11	CVA	2639	38	M	1	3	2	2	0	1	9
MCS 12	TBI	2690	24	M	3	3	5	1	0	2	14
MCS 13	TBI+anoxia	401	29	M	1	3	2	1	0	2	9
MCS 14	Anoxia	9900	39	M	3	3	5	2	0	2	15
MCS 15	Anoxia	396	57	M	3	0	2	2	0	2	9
MCS 16	TBI+Anoxia	314	26	F	3	1	2	1	0	2	9
MCS 17	TBI	407	31	M	0	1	2	2	0	1	6
MCS 18	Anoxia	64	29	M	1	3	2	2	0	2	10
MCS 19	Haemorrhage	242	46	F	2	3	2	1	0	2	10
MCS 20	Anoxia	639	43	M	2	3	1	2	0	2	10
MCS 21	TBI	1241	53	M	0	3	2	2	0	2	9
MCS 22	TBI	135	51	M	3	4	2	1	0	2	12
MCS 23	TBI	30	67	F	0	3	2	0	0	2	7
MCS 24	Haemorrhage	1383	68	F	3	1	3	2	0	2	11
MCS 25	TBI	1331	35	M	3	0	2	1	0	2	8
MCS 26	TBI - Haemorrhage	35	73	M	0	2	0	1	0	1	4
MCS 27	CVA	104	43	F	3	1	3	1	0	0	8
MCS 28	TBI	319	41	M	1	0	2	1	0	1	5
MCS 29	TBI - Haemorrhage	255	39	M	4	5	4	2	1	2	18
MCS 30	Anoxia	1482	32	M	3	4	2	2	1	2	14
MCS 31	TBI	641	23	M	3	3	0	1	0	2	9
MCS 32	TBI	37	26	F	2	3	3	0	1	1	10
MCS 33	Haemorrhage	389	59	F	2	1	2	1	0	2	8

Supplementary Table S7: **MCS patients' demographic and clinical characteristics.** The table includes condition, etiology (traumatic brain injury (TBI) and cerebral vascular accident (CVA)), time science injury (TSI), age, gender (F=female, M=male), Coma Recovery Scale-Revised (CSR-R) auditory, visual, motor, verbal, communication and arousal subscores and total.

Condition	Etiology	TSI	Age	Gender	Auditory	Visual	Motor	Verbal	Communication	Arousal	Total CRS-R
UWS 1	Anoxia	2890	49	M	1	1	1	2	0	2	7
UWS 2	TBI	283	52	F	1	0	2	2	0	1	6
UWS 3	Anoxia	743	30	M	1	0	2	1	0	2	6
UWS 4	Anoxia	92	74	M	1	0	1	1	0	1	4
UWS 5	Haemorrhage	43	64	M	1	0	2	1	0	1	5
UWS 6	Anoxia	18	20	M	1	0	0	1	0	1	3
UWS 7	Anoxia	1683	39	F	1	0	2	1	0	2	6
UWS 8	Anoxia	38	50	F	0	0	0	2	0	1	3
UWS 9	Anoxia	50	69	F	0	1	2	1	0	1	5
UWS 10	Anoxia	129	49	F	1	0	0	1	0	2	4
UWS 11	TBI	24	58	M	0	1	2	0	0	1	4
UWS 12	Anoxia	335	40	F	1	0	2	1	0	2	6
UWS 13	Anoxia	7814	34	M	1	0	1	1	0	2	5
UWS 14	Anoxic Asphyxia	304	60	M	1	1	1	1	0	2	6
UWS 15	Anoxia	30	44	M	1	1	1	1	0	1	5

Supplementary Table S8: **UWS patients' demographic and clinical characteristics.** The table includes condition, etiology (traumatic brain injury (TBI)), time science injury (TSI), age, gender (F=female, M=male), Coma Recovery Scale-Revised (CSR-R) auditory, visual, motor, verbal, communication and arousal subscores and total.

ROI	Corresponding label in AAL atlas	ROI	Corresponding label in AAL atlas
1	38 % Superior frontal gyrus, orbital part [6] / 25 % Middle frontal gyrus, orbital part [10]	55	67 % Inferior temporal gyrus [90]
2	42 % Gyrus rectus [28] / 19 % Olfactory cortex [22]	56	69 % Inferior temporal gyrus [90]
3	57 % Gyrus rectus [28] / 23 % Superior frontal gyrus, medial orbital [26]	57	67 % Inferior temporal gyrus [90]
4	69 % Superior frontal gyrus, orbital part [6]	58	48 % Fusiform gyrus [56] / 38 % Inferior temporal gyrus [90]
5	44 % Superior frontal gyrus, medial orbital [26] / 31 % Anterior cingulate and paracingulate gyri [32]	59	55 % Fusiform gyrus [56] / 41 % Inferior temporal gyrus [90]
6	34 % Superior frontal gyrus, dorsolateral [4] / 33 % Superior frontal gyrus, medial [24]	60	33 % Fusiform gyrus [56] / 27 % Inferior temporal gyrus [90]
7	41 % Middle frontal gyrus, orbital part [10] / 29 % Middle frontal gyrus [8]	61	50 % Superior temporal gyrus [82] / 28 % Rolandic operculum [18]
8	38 % Inferior frontal gyrus, orbital part [16] / 26 % Middle frontal gyrus, orbital par [10]	62	28 % Superior temporal gyrus [82] / 25 % Rolandic operculum [18]
9	61 % Middle frontal gyrus [8]	63	52 % Superior temporal gyrus [82] / 48 % Middle temporal gyrus [86]
10	60 % Superior frontal gyrus, medial[24] / 20 % Anterior cingulate and paracingulate gyri [32]	64	66 % Middle temporal gyrus [86]
11	84 % Middle frontal gyrus [8]	65	64 % Middle temporal gyrus [86]
12	52 % Superior frontal gyrus, dorsolateral [4] / 41 % Superior frontal gyrus, medial [24]	66	66 % Inferior temporal gyrus [90]
13	51 % Middle frontal gyrus [8] / 38 % Superior frontal gyrus, dorsolateral [4]	67	39 % Fusiform gyrus [56] / 25 % Inferior occipital gyrus [54]
14	85 % Middle frontal gyrus [8]	68	53 % Fusiform gyrus [56] / 16 % Lingual gyrus [48]
15	56 % Median cingulate and paracingulate gyri [34] / 26 % Anterior cingulate and paracingulate gyri [32]	69	51 % Inferior temporal gyrus [90] / 47 % Middle temporal gyrus [86]
16	45 % Inferior frontal gyrus, triangular part [14] / 28 % Inferior frontal gyrus, orbital part [16]	70	70 % Inferior temporal gyrus [90]
17	56 % Inferior frontal gyrus, orbital part [16] / 29 % Middle frontal gyrus, orbital part [10]	71	48 % Fusiform gyrus [56] / 29 %Inferior temporal gyrus [90]
18	59 %Inferior frontal gyrus, orbital part [16] / 20 % Insula [30]	72	51 % Lingual gyrus [48] / 33 % Fusiform gyrus [56]
19	67 % Inferior frontal gyrus, triangular part [14]	73	67 % Middle occipital gyrus [52]
20	57 % Insula [30] / 21 % Inferior frontal gyrus, triangular part [14]	74	34 % Middle occipital gyrus [52] / 32 % Middle temporal gyrus [86]
21	45 % Inferior frontal gyrus, opercular part [12] / 34 % Precental gyrus [2]	75	42 % Superior occipital gyrus [50] / 26 % Cuneus [46]
22	44 % Inferior frontal gyrus, opercular part [12] / 34 % Inferior frontal gyrus, triangular part [14]	76	48 % Lingual gyrus [48] / 17 % Fusiform gyrus [56]
23	74 % Postcentral gyrus [58]	77	65 % Cuneus [46]
24	57 % Supplementary motor area [20] / 39 % Paracentral Lobule [70]	78	37 %Middle occipital gyrus [52] / 20 % Superior occipital gyrus [50]
25	74 % Supplementary motor area [20]	79	78 % Lingual gyrus [48]
26	51 % Precental gyrus [2] / 43 % Superior frontal gyrus, dorsolateral [4]	80	39 % Calcarine fissure and surrounding cortex [44] / 27 % Cuneus [46]
27	81 % Precental gyrus [2]	81	59 %Inferior occipital gyrus [54] / 23 % Lingual gyrus [48]
28	60 % Supplementary motor area [20] / 22 % Median cingulate and paracingulate gyri [34]	82	76 % Calcarine fissure and surrounding cortex [44]
29	58 % Supplementary motor area [20] / 42 % Superior frontal gyrus, dorsolateral [4]	83	81 % Anterior cingulate and paracingulate gyri [32]
30	47 % Middle frontal gyrus [8] / 40 % Superior frontal gyrus, dorsolateral [4]	84	81 % Median cingulate and paracingulate gyri [34]
31	52 % Precental gyrus [2] / 22 % Inferior frontal gyrus, opercular part [12]	85	44 % Posterior cingulate gyrus [36] / 39 % Median cingulate and paracingulate gyri [34]
32	45 % Precental gyrus [2] / 25 % Middle frontal gyrus [8]	86	57 % Precuneus [68] / 32 % Calcarine fissure and surrounding cortex [44]
33	66 % Postcentral gyrus [58]	87	44 % Calcarine fissure and surrounding cortex [44] / 6 % Lingual gyrus [48]
34	66 % Insula [30]	88	42 % Median cingulate and paracingulate gyri [34]
35	56 % Insula [30] / 24 % Rolandic operculum [18]	89	80 % Median cingulate and paracingulate gyri [34]
36	52 % Insula [30] / 36 %Inferior frontal gyrus, orbital part [16]	90	79 % Precuneus [68]
37	34 % Insula [30] / 22 % Lenticular nucleus, putamen [74]	91	46 % Precuneus [68] / 37 % Median cingulate and paracingulate gyri [34]
38	47 % Postcentral gyrus [58] / 23 % Inferior parietal, but supramarginal and angular gyri [62]	92	28 % Amygdala [42] / 27 % Temporal pole: superior temporal gyrus [84]
39	63 % Postcentral gyrus [58]	93	50 % Hippocampus [38] / 9 % ParaHippocampal gyrus [40]
40	57 % Rolandic operculum [18] / 34 % Insula [30]	94	72 % Hippocampus [38]
41	70 % Superior parietal gyrus [60]	95	36 % ParaHippocampal gyrus [40] / 36 % Hippocampus [38]
42	49 % Precuneus [68] / 40 % Cuneus [46]	96	60 % ParaHippocampal gyrus [40] / 28 % Fusiform gyrus [56]
43	37 % Superior parietal gyrus [60] / 23 % Inferior parietal, but supramarginal and angular gyri [62]	97	67 % ParaHippocampal gyrus [40]
44	80 % Precuneus [68]	98	49 % Lingual gyrus [48] / 23 % Precuneus [68]
45	47 % Supramarginal gyrus [64] / 35 % Postcentral gyrus [58]	99	30 % Hippocampus [38] / 15 % Amygdala [42]
46	53 % Superior temporal gyrus [82] / 37 % SupraMarginal [64]	100	62 % Caudate nucleus [72]
47	46 % Supramarginal gyrus [64] / 34 % Inferior parietal, but supramarginal and angular gyri [62]	101	46 % Caudate nucleus [72] / 6 % Thalamus [78]
48	87 % Angular gyrus [66]	102	57 % Caudate nucleus [72] / 11 % Olfactory cortex [22]
49	73 % Middle occipital gyrus [52]	103	84 % Lenticular nucleus, putamen [74]
50	87 % Middle temporal gyrus [86]	104	19 % Lenticular nucleus, putamen [74] / 14 % Caudate nucleus [72]
51	56 % Temporal pole: middle temporal gyrus [88] / 20 % Fusiform gyrus [56]	105	36 % Thalamus [78] / 9 % Lingual gyrus [48]
52	62 % Temporal pole: middle temporal gyrus [88]	106	41 % Thalamus [78] / 8 % Hippocampus [38]
53	45 % Temporal pole: superior temporal gyrus [84] / 34 % Temporal pole: middle temporal gyrus [88]	107	52 % Thalamus [78]
54	58 % Middle temporal gyrus [86] / 32 % Superior temporal gyrus [82]		

Supplementary Table S9: **Correspondences of the Shen parcellation ROIs to the AAL atlas.**ROIs in the Shen atlas for one hemisphere (same parcellation for both hemispheres) and each correspondence to the AAL atlas. The percentage corresponds to the covered part of the ROI in the Automated Anatomical Labeling (AAL) parcellation code.

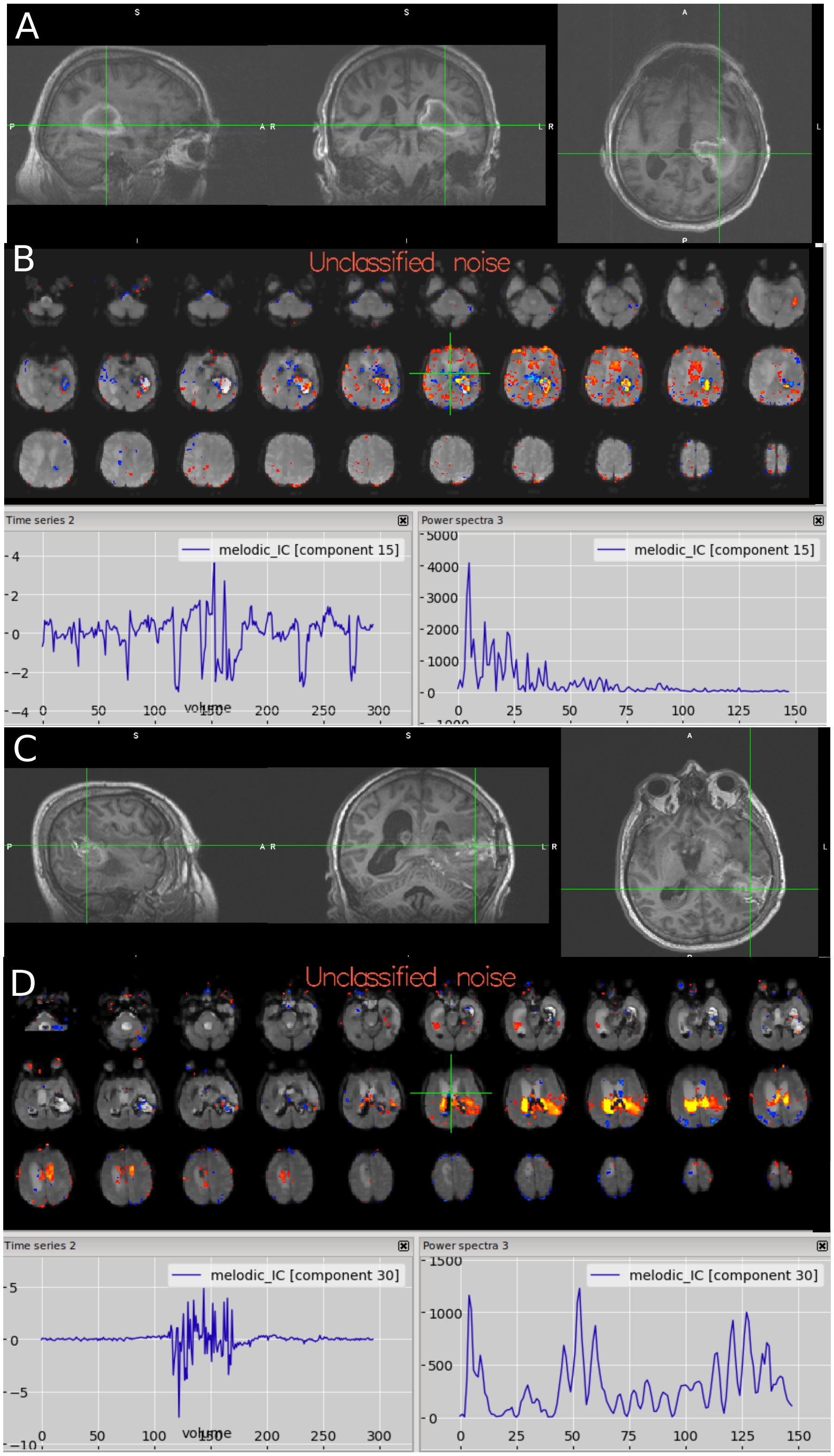

Supplementary Figure S10: **Examples of the lesion-driven artefacts cleaning based on melodic ICs components in the pre-processing. A and C)** Examples of the T1 weighted structural image of two UWS patients. T1 structural images show a localized lesion in the right cortex for each of the subjects. **B and D)** Components that show activation in the lesion. The high-resolution maps of the components show activation where the lesions are located. These components are considered noise, i.e. lesion-driven artefacts, so we classified them as "bad" components, and the FIX noisifier will exclude them.
