# Supplementary material for: Loss of consciousness reduces the stability of brain hubs and the heterogeneity of brain dynamics
Source: Commun Biol. 2021 Sep 6;4:1037. doi: 10.1038/s42003-021-02537-9 (PMC8421429; doi:10.1038/s42003-021-02537-9)
Supplement: Supplementary file 3 — Reporting Summary [file 42003_2021_2537_MOESM3_ESM.pdf]

nature research

Corresponding author(s): Ane López-González

Last updated by author(s): Jul 18, 2021

Reporting Summary

Nature Research wishes to improve the reproducibility of the work that we publish. This form provides structure for consistency and transparency in reporting. For further information on Nature Research policies, see our Editorial Policies and the Editorial Policy Checklist.

Statistics

For all statistical analyses, confirm that the following items are present in the figure legend, table legend, main text, or Methods section.

n/a	Confirmed	
☐	☒	The exact sample size (*n*) for each experimental group/condition, given as a discrete number and unit of measurement
☐	☒	A statement on whether measurements were taken from distinct samples or whether the same sample was measured repeatedly
☐	☒	The statistical test(s) used AND whether they are one- or two-sided<br>*Only common tests should be described solely by name; describe more complex techniques in the Methods section.*
☐	☒	A description of all covariates tested
☐	☒	A description of any assumptions or corrections, such as tests of normality and adjustment for multiple comparisons
☐	☒	A full description of the statistical parameters including central tendency (e.g. means) or other basic estimates (e.g. regression coefficient) AND variation (e.g. standard deviation) or associated estimates of uncertainty (e.g. confidence intervals)
☐	☒	For null hypothesis testing, the test statistic (e.g. *F*, *t*, *r*) with confidence intervals, effect sizes, degrees of freedom and *P* value noted<br>*Give P values as exact values whenever suitable.*
☒	☐	For Bayesian analysis, information on the choice of priors and Markov chain Monte Carlo settings
☐	☒	For hierarchical and complex designs, identification of the appropriate level for tests and full reporting of outcomes
☐	☒	Estimates of effect sizes (e.g. Cohen's *d*, Pearson's *r*), indicating how they were calculated

Our web collection on statistics for biologists contains articles on many of the points above.

Software and code

Policy information about availability of computer code

Data collection	Data will be avalaible in EBRAINS.
Data analysis	The codes for the data anaysis will be avalaible after publication (GitHub).

For manuscripts utilizing custom algorithms or software that are central to the research but not yet described in published literature, software must be made available to editors and reviewers. We strongly encourage code deposition in a community repository (e.g. GitHub). See the Nature Research guidelines for submitting code & software for further information.

Data

Policy information about availability of data

All manuscripts must include a data availability statement. This statement should provide the following information, where applicable:

- Accession codes, unique identifiers, or web links for publicly available datasets
- A list of figures that have associated raw data
- A description of any restrictions on data availability

The data can be requested to the Authors and examples of the phase-locking matrices of functional connectivity of different states of pharmacological and pathological states of consciousness are available on the Knowledge Graph (Human Brain Project). https://search.kg.ebrains.eu/live/minds/core/dataset/v1.0.0/775c7858-2305-4a56-8bd6-865c4ab5dd4f

Field-specific reporting

Please select the one below that is the best fit for your research. If you are not sure, read the appropriate sections before making your selection.

☒ Life sciences ☐ Behavioural & social sciences ☐ Ecological, evolutionary & environmental sciences

For a reference copy of the document with all sections, see nature.com/documents/nr-reporting-summary-flat.pdf

Life sciences study design

All studies must disclose on these points even when the disclosure is negative.

Sample size	No sample size calculation was performed.
Data exclusions	The exclusion criteria of patients were as follows: (i) having any significant neurological, neurosurgical or psychiatric disorders prior to the brain insult that lead to DOC, (ii) having any contraindication to MRI such as electronic implanted devices, external ventricular drain, and (iii) being not medically stable or large focal brain damage, i.e. >2/3 of one hemisphere.<br>The exclusions criteria of the healthy subjects that underwent anesthesia consist on having MRI contradication and any history of neurological or psychiatric disorders or drug consumption.
Replication	*Describe the measures taken to verify the reproducibility of the experimental findings. If all attempts at replication were successful, confirm this OR if there are any findings that were not replicated or cannot be reproduced, note this and describe why.*
Randomization	The diagnosis of the DOC patients was confirmed through repeated behavioural assessment with the Coma Recovery Scale-Revised (CRS-R) that evaluates auditory, visual, motor, sensorimotor function, communication and arousal.<br><br>For the propofol anesthedia, the level of consciousness was evaluated clinically throughout the Ramsay scale, representing the verbal commands.
Blinding	The preprocessing of DOC patients was performed blinded to the clinical diagnosis.

Reporting for specific materials, systems and methods

We require information from authors about some types of materials, experimental systems and methods used in many studies. Here, indicate whether each material, system or method listed is relevant to your study. If you are not sure if a list item applies to your research, read the appropriate section before selecting a response.

Materials & experimental systems

n/a	Involved in the study
☒	☐ Antibodies
☒	☐ Eukaryotic cell lines
☒	☐ Palaeontology and archaeology
☒	☐ Animals and other organisms
☐	☒ Human research participants
☐	☒ Clinical data
☒	☐ Dual use research of concern

Methods

n/a	Involved in the study
☒	☐ ChIP-seq
☒	☐ Flow cytometry
☐	☒ MRI-based neuroimaging

Human research participants

Policy information about studies involving human research participants

Population characteristics	We used two different datasets;<br><br>1- Disorder of consciousness (DOC): We selected 48 DOC patients, 33 in MCS (9 females, age range 24-83 years; mean age +/- SD, 45 +/- 16 years) and 15 with UWS (6 females, age range 20-74 years; mean age +/- SD, 47 +/- 16 years) and 35 age and gender-matched healthy controls (14 females, age range 19-72 years; mean age +/- SD, 40 +/- 14 years).<br><br>2- For the propofol anaesthesia, 16 healthy control subjects (14 females, age range, 18–31 years; mean age +/- SD, 22 +/- 3.3 years) were selected in three clinical states including normal wakefulness with eyes closed (W), anaesthesia-induced reduction of consciousness (S) and recovery from anaesthesia (R).
Recruitment	All the participants and patients were recruted in the hospital of Liège (Belgium).
Ethics oversight	Ethics Committee of the Faculty of Medicine of the University of Liège.

Note that full information on the approval of the study protocol must also be provided in the manuscript.

Clinical data

Policy information about clinical studies

All manuscripts should comply with the ICMJE guidelines for publication of clinical research and a completed CONSORT checklist must be included with all submissions.

Clinical trial registration: Faculty of Medicine of the University of Liège

Study protocol: The study protocol is not avalaible in this study, because it is deailed explained in the cited papers.

Data collection: The data was collected in the University Hospital of Liège (Belgium). The data was collected during 2010-2017.

Outcomes: The diagnosis of the DOC patients was confirmed through repeated behavioural assessment with the Coma Recovery Scale-Revised (CRS-R) that evaluates auditory, visual, motor, sensorimotor function, communication and arousal.

For the propofol anesthedia, the level of consciousness was evaluated clinically throughout the Ramsay scale, representing the verbal commands. The subject was asked to strongly squeeze the hand of the investigator. They were considered fully awake or to have recovered consciousness if the response to verbal command ("squeeze my hand") was clear and strong (Ramsay 2), in mild sedation, if the response to verbal command was clear but slow (Ramsay 3), and in deep sedation, if there was no response to verbal command (Ramsay 5–6).

Magnetic resonance imaging

Experimental design

Design type: Resting-state

Design specifications: One acquisition for participant or patient. In the case of DOC dataset, the acquisition consisted of 300 time points and the interval was of TR=2 (2 s).

In the case of propofol anesthesia, the acquisition consisted of 197 time points and the interval was of TR=2 (2 s).

Behavioral performance measures: Only resting-state.

Acquisition

Imaging type(s): Functional and structural

Field strength: 3 T

Sequence & imaging parameters: The BOLD fMRI resting state of the DOC patients was acquired using EPI, gradient echo with following parameters: volumes = 300, TR = 2000 ms, TE = 30 ms, flip angle = 78º, voxel size = 3 x 3 x3 mm^3, FOV = 192 x 192 mm^2, 32 transversal slices, with a duration of 10 minutes. Subsequently, structural 3D T1-weighted MP-RAGE images with were acquired with following parameters: 120 transversal slices, TR = 2300 ms, voxel size = 1.0 x 1.0x 1.2 mm^3, flip angle = 9º, FOV = 256 x 256$ mm^2.
The propofol dataset was acquired on a 3T Siemens Allegra scanner (Siemens AG, Munich, Germany). The fMRI resting state were acquired using the following parameters: EPI, gradient echo, volumes = 200; TR = 2460ms, TE = 40 ms, voxel size = 3.45 x 3.45 x 3 mm^3, FOV = 220x220 mm, 32 transverse slices, 64 x 64 x 32 matrix size.

Area of acquisition: Whole brain scan

Diffusion MRI: ☒ Used ☐ Not used

Parameters: DOC patients: Last, diffusion weighted MRI (DWI) was acquired in 64 directions (b-value =1,000 s/mm^2, voxel size = 1.8x1.8x3.3 mm^3, FOV = 230x230 mm^2, TR= 5,700 ms, TE= 87 ms, 45 transverse slices, 128x128 voxel matrix) preceded by a single unweighted image (b0).

Propofol dataset: The structural images were acquired using 3D T1-weighted MP-RAGE with following parameters: 120 transversal slices, TR = 2250 ms, TE=2.99ms, voxel size = 1 mm3, flip angle = 9º, FOV = 256 x 240 x 160mm.

Preprocessing

Preprocessing software: Preprocessing of MRI data was performed using MELODIC (Multivariate Exploratory Linear Optimized Decomposition into Independent Components) version 3.14, which is part of the FMRIB's Software Library (FSL). Preprocessing steps included: discarding the first 5 volumes, motion correction using MCFLIRT, non-brain removal using BET (Brain Extraction Tool), spatial smoothing with 5 mm FWHM Gaussian Kernel, rigid-body registration, high pass filter cutoff = 100.0 s, and single-session ICA with automatic dimensionality estimation.

Normalization: The cleaned functional data previously obtained were co-registered to the T1-weighted structural image by using FLIRT.

Normalization	Then, the T1-weighted image was co-registered to the standard MNI space by using FLIRT (12 DOF) and FNIRT.
Normalization template	The resulting transformations were concatenated and inverted and applied to warp the resting-state atlas from MNI space to the cleaned functional data.
Noise and artifact removal	FIX (FMRIB's ICA-based X-noiseifier) was applied to remove the noise components and the lesion-driven artefacts, independently, for each subject. Specifically, FSLeyes package in Melodic mode was used to manually classify the single-subject Independent Components (ICs) into "good" for signal, "bad" for noise or lesion-driven artefacts and "unknown" for ambiguous components. Each component was classified by looking at the spatial map, the time series, and the temporal power spectrum. Finally, FIX was applied by using the default parameters to obtain a cleaned version of the functional data.
Volume censoring	We performed the volume censoring applying the FIX-ICA noisefier.

Statistical modeling & inference

Model type and settings	NA
Effect(s) tested	one way ANOVA.

Specify type of analysis: ☒ Whole brain ☐ ROI-based ☐ Both

Statistic type for inference (See Eklund et al. 2016)	NA
Correction	Multiple comparison (FDR).

Models & analysis

n/a	Involved in the study
☐	☒ Functional and/or effective connectivity
☐	☒ Graph analysis
☒	☐ Multivariate modeling or predictive analysis

Functional and/or effective connectivity	We used functional connectivity and dynamical functional connectivity based on the phase differences among ROIs. BOLD phases were extracted in the 0.04-0.07 Hz frequency band using the Hilbert transform. This allows to obtain, at each time point t, a phase-interaction matrix given by the phase differences among the ROIs. We also evaluated the temporal recurrence of phase-interaction matrices over time, also known as the functional connectivity dynamics (FCD).
Graph analysis	We used weighted graphs given by the phase-interaction matrices. From that graphs we measured the global segregation, integration, phase-flutuations and functional connectivity dynamics.<br><br>We also analyzed the structural connectomes of the healthy controls and DOC patients using graph analysis. We used the weighted connectomes and we measured the degree of each ROI and the rich club organization.
